# Muscle regeneration after sepsis

**DOI:** 10.1186/s13054-016-1308-3

**Published:** 2016-05-19

**Authors:** Adrien Bouglé, Pierre Rocheteau, Tarek Sharshar, Fabrice Chrétien

**Affiliations:** Human Histopathology and Animal Models Unit, Infection and Epidemiology Department, Institut Pasteur, 75724 cedex15 Paris, France; Anesthesiology and Intensive Care, Institut de Cardiologie, Hôpitaux Universitaires La Pitié-Salpêtrière, Paris, 75013 France; Intensive Care, Hôpital Raymond Poincaré, Garches, 92380 France; Versailles Saint Quentin University, Versailles, 78000 France; TRIGGERSEP, F-CRIN Network, Versailles, 78000 France; Neuropathology Laboratory, Centre Hospitalier Sainte Anne, Paris, 75014 France; Paris Descartes University, Sorbonne Paris Cité, Paris, 75006 France

## Abstract

Severe critical illness is often complicated by intensive care unit-acquired weakness (ICU-AW), which is associated with increased ICU and post-ICU mortality, delayed weaning from mechanical ventilation and long-term functional disability. Several mechanisms have been implicated in the pathophysiology of ICU-AW, but muscle regeneration has not been investigated to any extent in this context, even though its involvement is suggested by the protracted functional consequences of ICU-AW. Recent data suggest that muscle regeneration could be impaired after sepsis, and that mesenchymal stem cell treatment could improve the post-injury muscle recovery.

The primary functions of skeletal musculature are locomotor activity, postural behavior, and breathing. Severe critical illness is often complicated by intensive care unit-acquired weakness (ICU-AW), which is clinically characterized by bilateral and symmetrical limb weakness and is related to a myopathy and/or axonal polyneuropathy. ICU-AW affects between 25 % and 60 % of patients mechanically ventilated for more than 7 days [1], and is associated with increased ICU and post-ICU mortality, delayed weaning from mechanical ventilation and long-term functional disability [2]. Most patients who develop ICU-AW have been admitted for a sepsis episode, and the main risk factors for ICU-AW include the severity of critical illness, immobilization, hyperglycemia, and the use of some medications, including steroids and neuromuscular agents, although this is somewhat controversial.

The pathophysiology of critical illness myopathy is thought to involve the following mechanisms: 1) impairment of muscular membrane excitability, secondary to a dysregulation of sodium channel gating [3]; 2) mitochondrial dysfunction leading to bioenergetic failure and oxidative stress [4]; and 3) proteolysis, mainly related to an activation of the ubiquitin-proteasome pathway [5]. These mechanisms can be triggered by various factors, notably systemic inflammatory mediators, endocrine dysfunction, immobilization, some drugs, and electrolyte disturbances. The protracted functional consequences of ICU-AW indicate that muscle regeneration is also impaired. Surprisingly, muscle regeneration, which essentially depends on the muscle stem cells (also called satellite cells (SC)), has not been extensively investigated in the context of critical illness. SC that are located at the periphery of the muscle fiber [6] are activated in response to any muscle injury and then proliferate and differentiate to repair or replace the damaged fibers, and also self-renew to replenish the muscle stem cell reservoir [7].

It was recently demonstrated in a murine model of polymicrobial peritonitis that SC activation, proliferation, and expression of myogenic markers were impaired after sepsis, leading to impaired muscle regeneration; however, the post-sepsis intramuscular administration of exogenous mesenchymal stem cells (MSCs) could reverse this SC dysfunction [8]. MSC treatment significantly improved the post-injury muscle recovery with decreasing necrosis and fibrosis but also increased the force of isolated single fibers. It is conceivable that a systemic anti-inflammatory effect of MSCs is involved, as their administration induced a decrease in the plasma levels of pro-inflammatory cytokines and procalcitonin. MSCs were previously shown to possess immunomodulatory effects via interaction with immune cells [9–11], the MSC secretome [12], and transfer of mitochondrial material [13]. Furthermore, MSC treatment enhances bacterial clearance during infections [14]. These different capabilities led researchers to test this treatment in two severe conditions frequently encountered in the ICU: acute respiratory distress syndrome (ARDS) and sepsis. A recently published review on 54 pre-clinical studies reported that treatment with MSCs could significantly decrease mortality in animals with acute respiratory failure [15]. MSC treatment has been tested in phase 1 trials [16], and clinical trials are on the way. Likewise, experimental studies in sepsis showed that MSC treatment after polymicrobial sepsis could reduce mortality and improve organ function [17], but also prevent the occurrence of muscle weakness or accelerate muscle recovery. One may argue that, beforehand, we need to: 1) make sure that critical illness myopathy is associated with SC dysfunction in patients; 2) understand why the sepsis has such a sustainable impact on SC which have been shown to resist anoxia up to 17 days after death [18]; and 3) investigate comprehensively the interactions between SC and MSCs, notably in the context of sepsis. The route and time of administration in patients must be addressed. Only a stepwise and comprehensive approach would allow us to determine whether or not MSCs are truly efficient, and also would enable us to identify new therapeutic targets.

## Abbreviations

ARDS: acute respiratory distress syndrome
ICU-AW: intensive care unit-acquired weakness
MSC: mesenchymal stem cell
SC: satellite cells
